# Ecotoxicological Studies on the Effect of Roundup^®^ (Glyphosate Formulation) on Marine Benthic Microalgae

**DOI:** 10.3390/ijerph18030884

**Published:** 2021-01-20

**Authors:** Zuzanna Sylwestrzak, Aleksandra Zgrundo, Filip Pniewski

**Affiliations:** Institute of Oceanography, University of Gdańsk, Al. M. Piłsudskiego 46, 81-378 Gdynia, Poland; aleksandra.zgrundo@ug.edu.pl (A.Z.); filip.pniewski@ug.edu.pl (F.P.)

**Keywords:** toxic effect, marine microphytobenthos, microalgal communities, Baltic Sea, algal growth inhibition test, ecotoxicological tests, glyphosate, Roundup^®^

## Abstract

Glyphosate is a very effective herbicide and the main active ingredient in Roundup^®^—the most extensively used herbicide in the world. Since glyphosate is highly water soluble it reaches water bodies easily in surface water runoff. This prompted us to undertake an experiment to evaluate the effects of glyphosate in Roundup^®^ on natural communities of marine microphytobenthos. Microphytobenthos communities were obtained from the environment, and after transporting them to the laboratory and acclimatizing them, they were tested under controlled conditions. Changes in microphytobenthos composition and structure and the deteriorating condition of the cells of community-forming organisms (assessed by analyzing changes in chloroplast shape) were used to assess the impact of Roundup^®^ on endpoints. The tests indicated that microphytobenthic communities were relatively resistant to herbicide. The species richness of the communities probably enabled them to rebuild effectively. Sensitive species were replaced by those more tolerant of glyphosate. Only at the highest glyphosate concentration (8.5 g·dm^−3^) tested was a strong negative effect noted that limited community abundance and eliminated some of the organisms. The dominant diatoms in the communities were replaced by intensively developing cyanobacteria, which ultimately comprised nearly 60% of all the cells observed in the communities.

## 1. Introduction

Glyphosate is the most extensively used herbicide in the world. It easily reaches water bodies through surface runoff waters and this affects photosynthetic microorganism communities that are often the foundation of the functioning of aquatic ecosystems. Hence, monitoring the reactions of microorganisms to glyphosate is an important element of environmental management [1]. Since relatively little is known about the impact glyphosate has on communities of aquatic organisms, and especially those of marine organisms, the aim of this study was to assess the impact of glyphosate in the popular herbicide Roundup^®^ on the condition of the natural microphytobenthos in the Baltic Sea.

Glyphosate is the active ingredient in non-selective herbicides that also contain many excipients, and it is an extremely effective compound that has a broad spectrum of biological activity. The popularity of the herbicide Roundup^®^, the main active ingredient of which is glyphosate, has grown with the proliferation of genetically modified crops [2]. Since it is believed that the combined effects of glyphosate and the expedients in products containing it are greater than those of glyphosate alone [3], we decided to assess the impact the product Roundup^®^ itself has on the environment. The increasing potential exposure of ever larger segments of society to glyphosate and the development of molecular test methods have both contributed to a growing interest in this substance in the context of its biological activity and glyphosate metabolites [4]. The concentration of glyphosate in European surface waters varies within the range of 0.67·10^−7^ g·dm^−3^ and 9·10^−6^ g·dm^−3^ depending on the sampling approach and measuring methods [5]. 

Chemically, glyphosate is N-phosphonomethyl glycine, which is highly hydrophilic. Its solubility is 10–15.7 g·L^−1^ at 25°, and its half-life in aquatic environments is less than seven days [6]. Herbicides containing the active ingredient glyphosate are very effective. After entering the plant, glyphosate inhibits the production of the enzyme EPSP synthase (5-enolpyruvylshikimate-3-phosphate). Inhibiting the activity of this enzyme prevents plants from forming the aromatic amino acids that are important for their growth and that are components of many plant pigments [7]. The reduced amount or lack of photosynthetic pigments affects the structure and functioning of chloroplasts [8]. Glyphosate also causes plant desiccation [9].

Humans release many chemicals into the seas, and these can cause varying degrees of environmental deterioration. Currently, much research is being conducted to expand knowledge about the state of and threats to the marine environment, and modern techniques used in water monitoring allow for the early detection of sources that threaten water bodies. Ecotoxicological studies are one of the tools used to assess the impact of chemical substances on aquatic environments. To date, most ecotoxicological studies have been performed on monocultures and single strains [10,11,12,13,14]. While these studies are extremely valuable, they provide information about the reaction of organisms only within the range of the so-called potential niche. Only studies that take into account whole communities make it possible to identify more reliably organism responses that reflect processes occurring in the environment, as they also include interactions among organisms. Furthermore, it has been shown that [15,16], microorganisms maintained as monocultures undergo microevolution changing their genetic makeup and thus phenotypic features. The evidence was also provided that “in-culture” evolution can finally led to establishing a strain optimally adapted to culturing conditions loosing adaptations typical of natural populations. Consequently, laboratory studies describe responses of altered organisms non-existing in the natural environment. Therefore, in our research we conducted toxicological tests on entire microphytobenthos communities obtained from the environment. Providing information on the response of the entire ecological assemblage to a toxic substance, such glyphosate and expedients from Roundup^®^ in high concentrations, will contribute to a better understanding of environmental responses to herbicides introduced by humans that have significant negative impacts on terrestrial plants and human itself.

## 2. Materials and Methods 

### 2.1. Field Work

**Figure 1:**
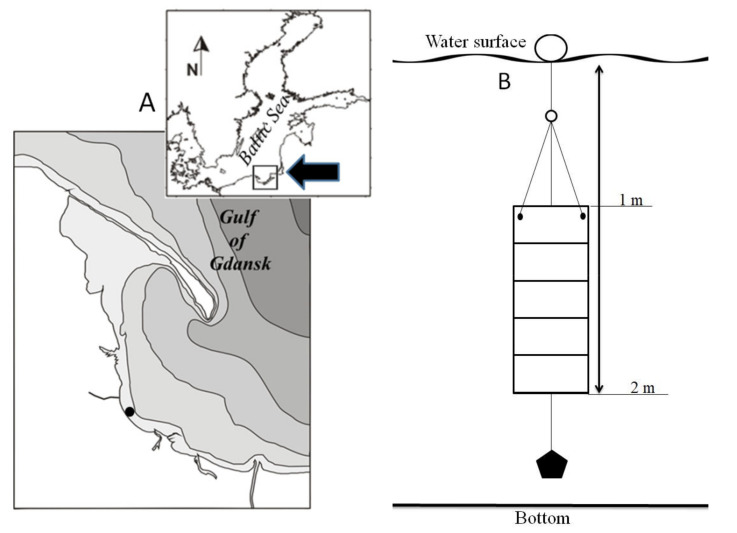
(**A**) Location of the station where the culture panel was exposed to the waters of the Gulf of Gdańsk. Inset shows the location of the Gulf of Gdańsk in the Baltic Sea. (**B**) Diagram of the culture panel exposed to the waters of the Gulf of Gdańsk.

Experiments on the impact glyphosate has on Baltic microalgae were conducted on microphytobenthic assemblages obtained from the environment. The microphytobenthos used in laboratory studies was obtained from glass slides exposed to the waters of the Gulf of Gdańsk (southern Baltic Sea) for a period of 14 days in the summer (Figure 1A). The water temperature during incubation was approximately 17–19 °C, and salinity ranged from 7.9 to 8.4 PSU. The culture panel (100 × 40 × 10 cm) with microscope slides (76 × 26 × 1 mm) was deployed at a depth of approximately 1–2 m (Figure 1B) at a distance of about 300 m from the shore at a station located at 54°26′49″N, 8°34′24″E (Figure 1A). The panel with the microscope slides on which microphytobenthos had grown were immediately placed in large containers filled with sea water collected in situ so that the slides remained immersed in the water. These were transported carefully so they reached the laboratory undisturbed. Transport time was about 15 min, and laboratory work began immediately upon their delivery. 

### 2.2. Preparation of Microalgal Suspensions and Experiment Design

After transporting the panels to the laboratory, the microphytobenthic communities growing on the microscope slides were scraped off with a scalpel. The microphytobenthos collected were sonicated with an impulse force that permitted mixing the cells thoroughly but avoided weakening or destroying them [Pniewski, unpublished research]. After sonication for a period of two minutes, the microphytobenthos was placed in 250 mL flasks in 100 mL of sea water collected in situ and filtered with Whatman GF/C filters and subsequently autoclaved. The natural concentrations of the nutrient compounds in sea water were: N-NH_4_ 9.4 mg·m^−3^, N-NO_3_ 102 mg·m^−3,^ P-PO_4_ 36 mg·m^−3^, Si-SiO_4_ 600 mg·m^−3^. With such high nutrient values, the decision was made not to add the culture medium to the solution because the additional nutrients would not have been used by the organisms within seven days. Additionally, excess nutrients could have caused additional stress for the organisms that comprised the microphytobenthos communities, which was something we wanted to avoid. Flasks with algal suspensions were saturated with nitrogen for 30 s to eliminate animal microorganisms following the standard method [17]. Then the communities were acclimated in a thermostatic chamber for a period of 72 h. The initial mean abundance of microalgal cells in flasks calculated was as high as 38,800 cells/mL ± 700.

After the acclimation period, the microphytobenthos was subjected to glyphosate toxicity tests as follows: control—microphytobenthos assemblage in 100 mL filtered seawater; test solutions—microphytobenthos assemblage in 100 mL filtered seawater at three solution of glyphosate (Roundup^®^) of 0.042 g·dm^−3^, 0.85 g·dm^−3^, and 8.5 g·dm^−3^. All experimental variants were performed in three replicates. The glyphosate concentrations were selected based on current literature on the subject and results of previously carried out experiments [8,13,18,19,20,21]. The lowest concentration of glyphosate (0.042 g·dm^−3^) was selected based on previously published values shown to cause inhibitory effects in microalgal strains typically used for ecotoxicological tests [8,17]. The concentration of 0.85 g·dm^−3^ was selected as a dose having weak but still noticeable effect on microphytobenthic communities [19,21]. The highest glyphosate concentration (8.5 g·dm^−3^), was chosen based on the results of preliminary experiments carried out on the Baltic microphytobenthic communities and indicated as having a substantial influence on the species composition [18,20].

### 2.3. Microscopic Analysis

**Figure 2:**
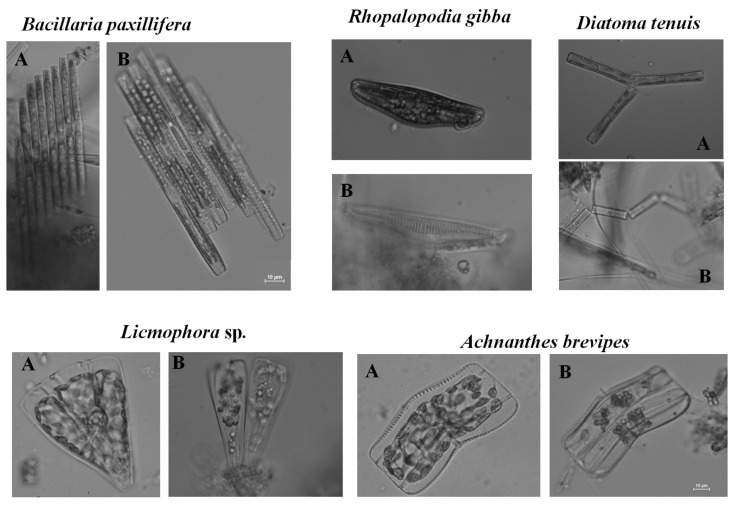
Cells of selected taxa with normal chloroplasts (A) and with abnormal chloroplasts (B).

Qualitative and quantitative changes in assemblage structure, i.e., changes in taxonomic composition and taxon abundance, were the primary parameters used to assess the changes in the microphytobenthos. Observations of the assemblages preserved in Lugol solution were performed in triplicate after one, three, and seven days for 50 fields of vision in sedimentation chambers (2 mL) under an Eclipse TS100 inverted light microscope (Nikon, Tokyo, Japan at magnifications of ×200 and ×400 according to principles in Organisation for Economic Co-operation and Development (OECD) guidelines for assessing the effects of chemical toxicity on plant microorganisms [22]. In each field of vision all cells were counted and identified. Cell numbers were counted according to the Utermöhl method [23] and Helcom [24] guidelines in which units are considered to be cells or threads at 100 μm in length. The microalgae were identified using [25,26,27,28,29,30,31,32]. Additionally, analysis of the condition of microalgal cells occurring in the microphytobenthos were conducted in three replicates after one, three, and seven days by observing the state of the chloroplasts in all cells present in 50 fields of vision under a Nikon Eclipse 80i microscope fitted with a Nikon DSU2 camera at a magnification of ×400. Observations were conducted based on previously developed research methodology [8,18]. During the observations, three groups of cells were identified: live cells with normal chloroplasts, live cells with abnormal chloroplasts, and dead cells. In this article, only the results for the cell groups with normal and abnormal chloroplasts are presented (Figure 2).

### 2.4. Statistical Analysis 

The data obtained were processed with MS Excel. Student’s t-test was performed to compare the significance of differences in cell numbers between glyphosate concentrations and the control solution and to designate differences among successive test days with STATISTICA version 10 (StatSoft, Inc., Tulsa, OK, USA). 

## 3. Results

### 3.1. Quantitative and Qualitative Analysis of Assemblages

During the experiment, a total of 58 microalgae species was identified, including 45 diatom, nine cyanobacterium, and two green alga taxa and representatives of Myzozoa (*Peridinium* sp.) and Haptophyta (*Prymnesium* sp.). The full list of taxa identified is in Appendix A (Table A1). Appendix A (Table A2) presents the mean abundance and standard deviation of selected taxa.

**Figure 3:**
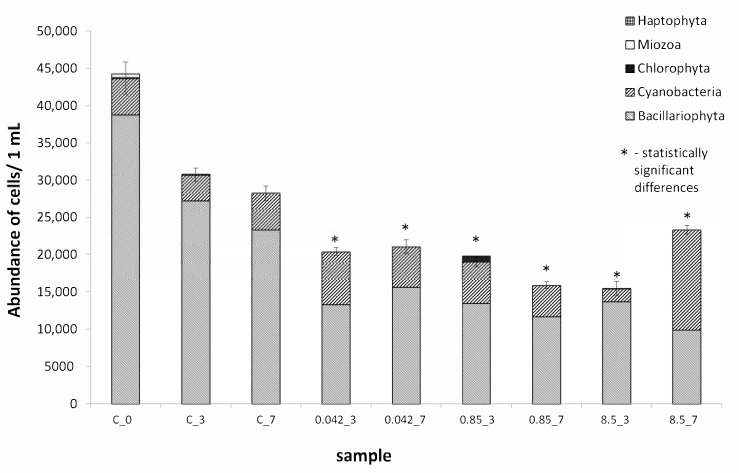
Abundance of microalgae in 1 mL suspension in control solutions: C_0—the start of the experiment; C_3—after three days of the test; C_7—after seven days of the test; and in the concentrations of glyphosate tested: of 0.042 g·dm^−3^ glyphosate (0.042_3) after three and (0.042_7) after seven days of tests; 0.85 g·dm^−3^ glyphosate (0.85_3) after three and (0.85_7) after seven days of tests; 8.5 g·dm^−3^ glyphosate (8.5_3) after three and (8.5_7) after seven days of tests. Statistically significant differences were denoted with the asterisk.

The highest mean abundance of 44,000 cells ± 2000 was observed at the beginning of the tests (Figure 3). Surprisingly, differences in cell abundance on days three and seven in the 0.042 g·dm^−3^ glyphosate solution were statistically insignificant and almost identical at 46% and 47% of the control samples, respectively. The smallest abundance was observed after day three in the concentration of 8.5 g·dm^−3^ glyphosate at 50% of the control cell abundance (statistically significant difference, *p* > 0.05), but at this concentration the number of cells increased to 82% of the control value on day seven of the tests.

The microphytobenthos assemblages analyzed were dominated by diatoms, which constituted from 65 to 88% of all the cells counted (in the control solution on day three). Only on day seven at a concentration of 8.5 g·dm^−3^ did they account for 43% of total abundance. The abundance of cyanobacteria in the control solution did not exceed 18% (the most were observed on day seven of the experiment), while at concentrations of 0.042 g·dm^−3^ and 0.85 g·dm^−3^ they constituted from 26 to 35% of total abundance. The most cyanobacteria were observed on day seven of the experiment in the solution at a concentration of 8.5 g·dm^−3^ at 57%.

### 3.2. Abundance of Selected Taxa

**Figure 4:**
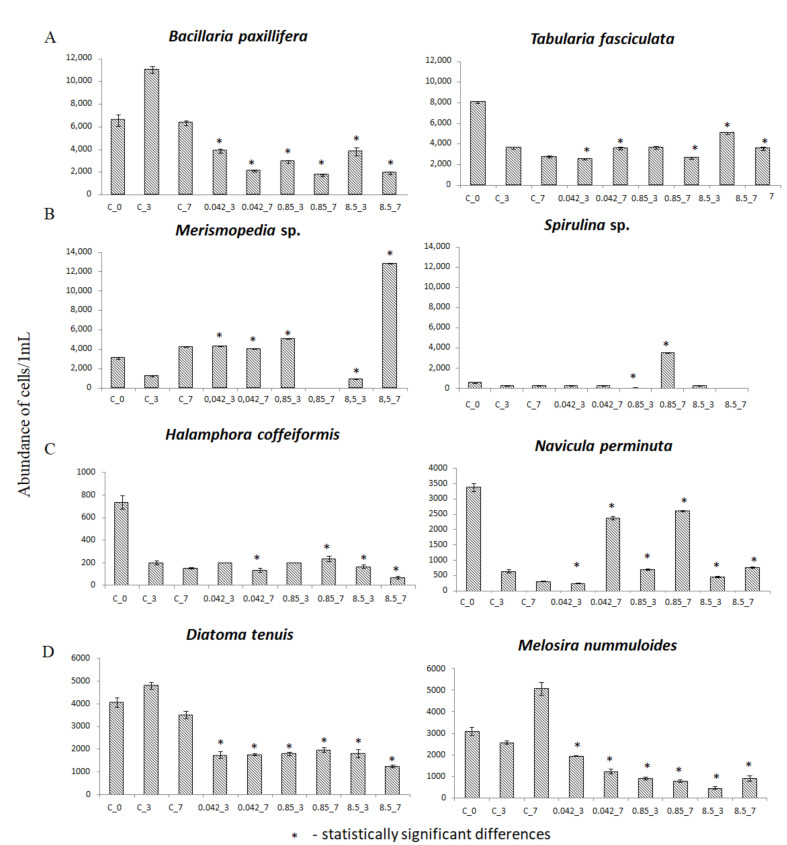
Abundance of cells of selected taxa in control solution: C_0—the start of the experiment; C_3—after three days of the test; C_7—after seven days of the test; and in glyphosate concentrations of glyphosate tested of: 0.042 g·dm^−3^ glyphosate (0.042_3) after three and (0.042_7) after seven days of tests; 0.85 g·dm^−3^ glyphosate (0.85_3) after three and (0.85_7) after seven days of tests; 8.5 g·dm^−3^ glyphosate (8.5_3) after three and (8.5_7) after seven days of tests. Statistically significant differences were denoted with the asterisk. (**A**)—dominant species on which glyphosate had a negative effect on growth, (**B**)—cyanobacteria in which glyphosate stimulated growth selectively, (**C**)—resistant species on which glyphosate had a positive effect on abundance, (**D**)—sensitive species on which glyphosate had a negative effect on abundance.

The microphytobenthic communities tested were dominated by diatoms such as *Tabularia fasciculata* and *Bacillaria paxillifera* that were 18 and 17%, respectively, of the entire community at the start of the experiment. The abundance of *T. fasciculata* on day 0 was 8000 ± 93 cells/1 mL) (Figure 4A). On subsequent days of the experiment in the concentration of 0.85 g·dm^−3^ glyphosate, no significant differences were noted in changes in the number of cells of this taxon (1% more on day three and 4% less on day seven in comparison to the control solution). In the 0.042 g·dm^−3^ solution 30% fewer cells than in the control solution were observed on day three, but by day seven there were 30% more. A different reaction was observed in the highest concentration tested, and on day three abundance was observed to increase by 40%, and on day seven there were 29% more cells in the control solutions. Increases in *B. paxillifera* abundance of 69% were observed in the control solution on day three, but on day seven abundance was once again similar to that at the start of the experiment. The abundance of this species decreased in comparison to the control from 65 to 74% in all of the concentrations tested on day three and on day seven from 67 to 73% (Figure 4A). Representatives of cyanobacteria *Merismopedia* sp. contributed a large share to the communities tested; at the beginning of the experiment *Merismopedia* sp. cells were 7% of all the those observed. An increase in the numbers of these cyanobacterium cells of 27% was observed in the control solution on day seven of the experiment (Figure 4 B). The presence of glyphosate had a stimulatory effect on the growth of the numbers of *Merismopedia* sp. cells, for example, the number of cells increased by approximately four times on day three at concentrations of 0.042 g·dm^−3^ and 0.85 g·dm^−3^. In the highest concentration tested of 8.5 g·dm^−3^ on day seven three times the number of *Merismopedia* sp. cells were observed than in the control. On the other hand, the number of units of other cyanobacterium of the genera *Spirulina* was small and did not differ in either the control or in the glyphosate solutions throughout the experiment (except for the cultures exposed to the glyphosate concentration of 0.85 g·dm^−3^ at which on day three the cell number was twice as low compared to the control solution). However, on day seven of the experiment at the concentration of 0.85 g·dm^−3^ an increased number of cells of this species was observed at 1750% of the control values (Figure 4B). *Halamphora cofeiformis* was identified as a tolerant species since small changes in numbers were observed at low glyphosate concentrations, for example, on day three numbers of it were comparable to those observed in the control solution. Only at a concentration of 8.5 g·dm^−3^ on day seven was a decrease observed in the number of cells to 56% fewer than in the control solution (Figure 4C). Some species, such as *Navicula perminuta*, turned out to be resistant to the applied Roundup^®^ concentrations and after an initial decrease, on day seven the cell number significantly increased. At concentrations of 0.042 g·dm^−3^ and 0.85 g·dm^−3^ growth was seven and eight times, respectively, higher in comparison to the control (Figure 4C). During the experiment, decreases in the numbers of cells of especially sensitive species, such as *Diatoma tenuis*, were noted in all concentrations by approximately 40% in comparison to the control solution; however, no large differences in numbers were observed among concentrations or on subsequent days of the tests (Figure 4D). Results were similar for *Melosira nummuloides* in which the highest numbers (24% fewer cells than in the control solution) were observed on day three at the concentration of 0.042 g·dm^−3^, while on day seven the number of cells decreased by 76%. At the concentration of 0.85 g·dm^−3^ from 65 to 85% fewer cells were noted than in the control solution, and there were 82% fewer cells of this taxon at the highest concentration tested. 

### 3.3. Cell Condition in Selected Taxa

**Figure 5:**
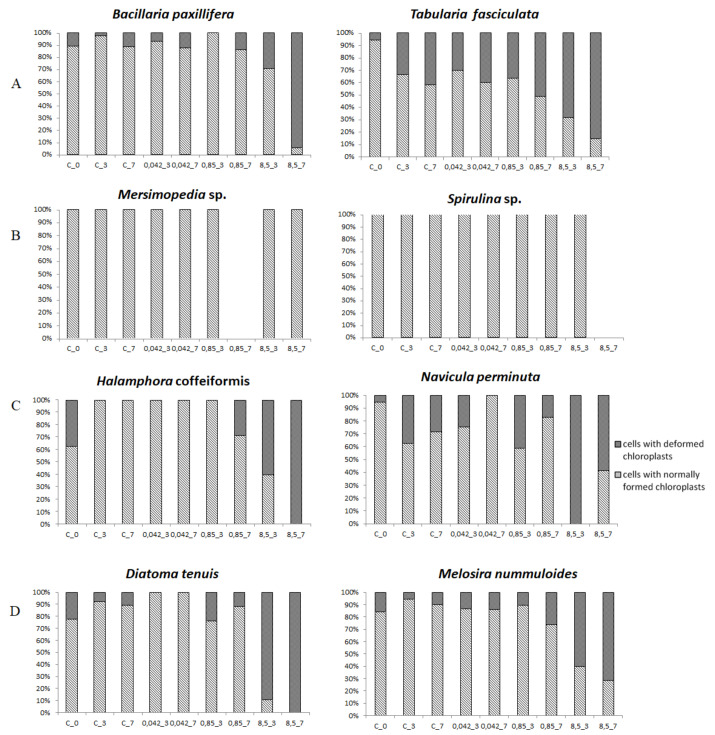
Changes in the condition of selected species expressed as the percentage share of cells with normal chloroplasts and abnormal chloroplasts in the control solution (**C**) and in the tested glyphosate solutions (0.042 g·dm^−3^, 0.85 g·dm^−3^, and 8.52 g·dm^−3^) at the start of the experiment and after three and seven days of the tests. (**A**)—dominant species on which glyphosate had a negative effect on growth, (**B**)—cyanobacteria in which glyphosate stimulated growth selectively, C—resistant species on which glyphosate had a positive effect on abundance, (**D**)—sensitive species on which glyphosate had a negative effect on abundance.

The analysis of chloroplast condition in selected taxa indicated differences among the main dominants. *Bacillaria paxillifera* cells were in good condition, and the number of cells with damaged chloroplasts was small and did not exceed 15% of live cells at most concentrations. Only at the concentration of 8.5 g·dm^−3^ was chloroplast degradation noted in 30% of live cells on day three and in 94% on day seven. In *Tabularia fasciculata* degraded chloroplasts were observed in 30 to 40% of live cells in the control on days three and seven and at concentrations of 0.042 g·dm^−3^ and 0.85 g·dm^−3^. On the other hand, at a concentration of 0.85 g·dm^−3^ on day seven, 45% of cells had abnormal chloroplasts, while as many as 85% did in the concentration of 8.5 g·dm^−3^ (Figure 5A). Among the cyanobacteria *Merismopedia* sp. and *Spirulina* sp. all cells examined appeared to be normal. Among the diatom species identified as resistant to the effects of glyphosate, such as *Halamphora coffeiformis* and *Navicula perminuta*, cells with deformed chloroplasts were less than 60% with the exception of cells in the concentration of 8.5 g·dm^−3^ (up to 100%). Interestingly, *N. perminuta* cells were in worse condition than, for example, those of *T. fasciculata* on day three of the experiment (Figure 5C). Evident effects of glyphosate on chloroplasts were observed in both sensitive (*Diatoma tenuis*, *Melosira nummuloides*) and resistant diatoms mainly at the concentration of 8.5 g·dm^−3^. 

## 4. Discussion

Toxicological tests on microalgae (most commonly planktonic marine diatoms) are conducted widely throughout the world [1,10,33,34]. Many of these tests are conducted on single strains, which only provide information regarding so-called potential niches. Only research that takes into consideration entire assemblages make it possible to obtain a more reliable picture of the reactions that occur among the taxa tested. To date, most tests conducted on glyphosate toxicity have focused on organisms inhabiting fresh waters [1,14], but our study concentrated on marine and brackish water organisms forming microphytobenthic communities in the southern Baltic (Gulf of Gdańsk). In our opinion, this is particularly interesting because marine coastal zones are the main areas that receive most of the pollution from surface water runoff. For example, the Gulf of Gdańsk, which is the area from which the communities used in the study were collected, is the basin that receives the waters of Poland’s largest rivers, including the Vistula that collects pollutants from a surface area of 193,960 km^2^, which is more than half of the country [35]. Large quantities of glyphosate potentially reach the Baltic, but studies of glyphosate in water bodies are not conducted widely. A few researchers have performed such studies, and their results confirm that there is glyphosate in open waters [5,36]. Researchers have studied the content of glyphosate and aminomethylphosphonic acid (AMPA) (the main glyphosate metabolite) in river mouths in the Baltic Sea and revealed that there was glyphosate in most of them at concentrations ranging from 2.8·× 10^−8^ g·dm^−3^ to 9·× 10^−5^ g·dm^−3^, while APMA was detected in all of them. While the half-life of glyphosate in aquatic environments under aerobic conditions is less than seven days [6], in soils its half-life ranges from two to 174 days [37]. The recommended single dose for resistant weeds is 21.6 g 100 m^2^, Coupe and coworkers [38] suggested that with the water runoff form agriculture areas even (up to) 0.86% of a Roundup^®^ dose can be directly transported into the surface waters. Thus, the concentrations used in our study can correspond to concentrations of glyphosate after applying the herbicide Roundup^®^. Intense precipitation, river runoff, and the fact that this compound can accumulate in soils for as many as 174 days can lead to very high concentrations of glyphosate in water bodies. 

Glyphosate affects plant growth by inhibiting the production of aromatic amino acids that halts the production of protein [39]. It also inhibits the production and activity of the enzyme 5-enolpyruvylshikimate-3-phosphate (EPSP) synthetase [40] that halts the synthesis of compounds that are important for plant growth, such as phenylalanine, tyrosine, and tryptophan, which are found in many plant pigments, flavonoids, and anthocyanins [7]. The reduced amounts or lack of photosynthetic pigments has a negative impact on the structure and functioning of chloroplasts [18]. Additionally, Roundup^®^ contains surfactants such as isopropylamine salt (IPA) and polyoxyethylene amines (POEA) that are added to the product to increase its effectiveness [1,41]. Surfactants are often considered manufacturer trade secrets, and the precise chemical compositions and actions of these compounds are currently unknown. Therefore, only studying the effects of Roundup^®^ as a product can answer questions about how it affects organisms and the impact it has on them. 

Based on the qualitative and quantitative analyses of natural microphytobenthos communities, several groups of microalgae were identified that were the most important. Diatoms were the most abundant in communities in the control solution, they constituted, on average, about 80% of the entire community. Cyanobacteria were the second most frequently observed group of microorganisms in each sample. The remaining groups of microorganisms contributed small shares to the communities and were not permanent elements of the communities studied. Their abundance did not exceed 1.5%. An interesting relationship between diatoms and cyanobacteria was observed in the glyphosate solution at a concentration of 8.5 g·dm^−3^; the number of cyanobacterial cells increased substantially between days three and seven. Our observations are reflected in the research of other authors. For example, Forlani [42] showed that some cyanobacteria could break down glyphosate into simpler compounds and then use them as a source of phosphorus, which might contribute to rapid increases in their numbers. These glyphosate properties and its long half-life in soils (up to 174 days) could contribute to increased concentrations of it in water bodies. Following weed suppression in spring, heavy precipitation and surface water runoff into rivers transports this substance to water bodies where it can be used as a source of phosphorus and carbon thus contributing to cyanobacterial blooms whose harmful effects are widely known. This assumptions is reflected in studies on the toxicity of various products in which the active ingredient is glyphosate. Gonzalez [1] observed increased cyanobacterial numbers and decreased numbers of *Chlorophyta* and *Bacillariophyta* in all tests performed using glyphosate at a concentration of just 0.003 g·dm^−3^. In turn, Berman [43] observed large quantities of picoplanktonic cyanobacteria in post-agricultural areas, which were linked to the glyphosate in their soils and waters. In our tests on marine microphytobenthic communities, the intensive development of cyanobacteria (e.g., *Merismopedia* sp., *Spirulina subsalsa*) in higher glyphosate concentrations could have been caused by increased amounts of phosphorus, nitrogen, or carbon that these organisms could have obtained from glyphosate. It is interesting that during our research we observed a significant increase in the number of *Spirulna* sp. cells on day seven in the concentration of 0.85 g·dm^−3^ that replaced the cells of another cyanobacterium—*Merismopedia* sp.

The analysis of the structure of communities exposed to glyphosate indicated that some groups of organisms were particularly resistant to this substance (e.g., *Merismopedia* sp., *Navicula perminuta*, *Entomoneis paludosa*), but there were also many other organisms in which rapid chloroplast degradation and cell growth inhibition were noted (e.g., *Navicula ramosissima*, *Diatoma tenuis*) (Table A2). The Roundup^®^ safety data sheet states that the EC_50_ value for *Selenastrum capricornutum* for 72-h exposure is 0.014 g·dm^−3^ [37]. Glyphosate toxicity tests on *Scenedesmus quadricauda* indicated that small glyphosate concentrations (0.002 g·dm^−3^) stimulated photosynthesis and chlorophyll *a* synthesis [11]. In our tests in a concentration trpile as high of 0.042 g·dm^−3^ on, for example, we observed increased numbers of cells in the cyanobacterium *Merismopedia* sp. implying that conditions were favorable for this organism, which meant more intense photosynthesis and chlorophyll *a* synthesis. As previously mentioned, reports in the literature indicate that Roundup^®^ can be a source of carbon or nitrogen, and low concentrations of it can stimulate microalgal cell growth [44,45]. On the other hand, Sáenz et al. [46] revealed that at a concentration of 0.1 g·dm^−3^ it caused the total cessation of the growth of the green alga *S. quadricauda* after 96 h of exposure. In turn, Hernando [47] reported that tests conducted over seven days on *Chlorella pyrenoidosa*, another green alga species, indicated that this alga is more resistant to the effects of Roundup^®^ since the EC_50_ for this species is 0.189 g·dm^−3^. In our study we showed that the cells of green algae of the genus *Scenedesmus* were present on day seven of the tests at all tested concentrations (data not presented), which suggested that even very high concentrations of glyphosate did not eliminate these taxa from microorganism communities. In laboratory ecotoxicological studies conducted on the diatom species *B. paxilifera* isolated from the Baltic Sea, it was shown that a glyphosate concentration of 0.05 g·dm^3^ caused a decrease in cell numbers of 51% after seven days in comparison to control conditions [13]. However, in our experiment on communities we observed growth inhibition of *B. paxilifera* on day seven of the tests of 22% at a concentration of 0.042 g·dm^−3^ and of 23% of the control value at a concentration of 0.85 g·dm^−3^, while at a concentration of 8.5 g·dm^−3^ we observed 51% fewer cells than in the control solution. It is an interesting fact that at lower concentrations despite substantial growth inhibition few damaged chloroplast cells were noted (12 and 14%, respectively). Differences were noted with the concentration of 8.5 g·dm^−3^ in which abnormal chloroplasts were observed in as many as 94% of *B. paxilifera* cells. Because the life cycles of aquatic microorganisms are short (from one to several days), ecotoxicological studies often represent chronic toxicity even with relatively short periods of exposure of three to five days [48]. While the manufacturers of Roundup^®^ indicate that the effects of the product are evident in target plants within seven to 10 days (yellowing and desiccation are noted that indicate, i.e., chloroplast degradation), plant death does not occur for up to three weeks [37].

Studies conducted on communities of freshwater periphyton indicated that natural communities adapted to stress factors, such as toxic substances, by altering community structure through the robust development of cyanobacteria and the inhibition of diatom growth [49]. Our observations in the current experiment were identical. Additionally, during the study we also examined the condition of chloroplasts, which indicated that usually advanced degradation only occurred at the highest concentration of 8.5 g·dm^−3^ (i.e., in *Halamphora coffeaeformis*, *Bacillaria paxilifera*, *Diatoma tenuis*, and *Melosira nummuloides*). 

Based on the tests we performed, the microphytobenthos communities were relatively resistant to glyphosate. Their high species variability meant that they were able to rebuild communities. Sensitive species were replaced by ones that were more resistant to glyphosate or were able to break down glyphosate and use this compound as a source of essential nutrient salts [42]. Tsui and Chu [41] showed that the toxicity of Roundup^®^ might not only stem from the glyphosate content, but also from isopropylamine (IPA) salts and polyoxyethylene amines (POEA). In studies of microphotoautotrophs from marine or freshwater communities, Lipok et al. [50] demonstrated that IPA salts were more toxic than glyphosate. Although algae are more susceptible to the herbicidal effect of IPA and glyphosate salts than non-photosynthetic organisms, they are able to activate defense mechanisms so they can survive in environments in which these compounds occur. Algae have similar metabolic pathways to higher plants (e.g., they synthesize aromatic amino acids), which also makes them susceptible to glyphosate [41]. However, as already mentioned, some species, such as some cyanobacteria, can use the substances into which glyphosate decomposes. The second strategy is the robust development of resistant or tolerant organisms that can occupy the vacant niches of organisms that glyphosate eliminates. In studies conducted on monocultures in fresh and brackish waters, Tsui and Chu [41] demonstrated that not until the concentration of glyphosate (from Roundup^®^) was as high as 7.2 g·dm^3^ was microbiological life totally destroyed. Despite the changes in structure we observed in the present experiment, the communities were able to function in the concentration of 8.5 g·dm^3^ glyphosate, and their numbers were smaller than the control by approximately 50% on day three and 20% on day seven. Concentrations observed in the environment to date [36] are many times lower, but even they have an impact on microalgae functioning in communities. The breakdown of glyphosate produces nutrients that enrich the environment by stimulating growth in many microalgal species. Therefore, even small concentrations of glyphosate that reach the environment can disrupt species equilibrium by stimulating the growth of selected taxa, and not, as might be expected, by reducing the number of many species. However, even high concentrations (8.5 g·dm^3^) did not cause substantial degradation in the relatively rich community we studied (58 taxa), but only caused it to rebuild with the robust development of cyanobacteria. Harmful algal blooms form an increasing problem in many aquatic environments, both freshwater and marine. The massive development of cyanobacteria in the Baltic Sea is associated with increasing eutrophication. While several bloom-forming algae are toxic, non-toxic algal blooms can also have a negative impact on the environment, as depletion of O_2_ and formation of toxic sulfide during bloom decaying, leading to degradation of many elements of ecosystem [51,52].

The results obtained here can be applied to all marine regions with similar salinity or trophic conditions as all the species identified in the studied assemblages are cosmopolitan and observed not only in the waters of the Gulf of Gdansk and the Baltic Sea [53,54,55,56], but also in the world oceans [30,57].

## 5. Conclusions

Tests performed on microphytobenthic communities revealed that they were relatively resistant to the effects of the glyphosate in Roundup^®^. The species richness of the communities permitted them to rebuild quickly and effectively by replacing sensitive species with tolerant and resistant ones. Organisms were divided into three groups depending on their reactions: those that were neutral to the effects of glyphosate (e.g., *Tabularia fasciculata*, *Halamphora coffeaeformis*), those that were stimulated by glyphosate (cyanobacteria such as *Merismopedia* sp. and *Spirulina* sp. and diatoms such as *Navicula perminuta*), and those that were sensitive to the presence of glyphosate (*Bacillaria paxilifera*, *Diatoma tenuis*, *Melosira nummuloides*). High glyphosate concentrations of 8.5 g·dm^−3^ had a negative impact on some organisms and strongly limited diatom growth. However, even this high concentration was preferred by some organisms and facilitated the mass development of cyanobacteria, which dominated the communities by day seven. The analysis of chloroplast condition indicated that their advanced degradation was usually noted at the highest concentration of 8.5 g·dm^−3^ (e.g., *Halamphora coffeaeformis*, *Bacillaria paxilifera*, *Diatoma tenuis*, *Melosira nummuloides*). 

## Data Availability

Open access Creative Commons CC BY 4.0 license.

## Appendix A

**Table A1:** List of taxa identified in the material studied.

Group of Algae	Taxon	Author
*Bacillariophyta*	*Achnanthes brevipes*	Bory
	*Achnan* *thes lemmermannii*	Hustedt
	*Amphora ovalis*	(Kützing) Kützing
	*Amphora pediculus*	(Kützing) Grunow
	*Amphora* sp.	Kützing
	*Bacillaria paxillifera*	(O.F.Müller) T.Marsson
	*Berkeleya rutilans*	(Trentepohl ex Roth) Grunow
	*Brebissonia lanceolata*	(C.Agardh) R.K.Mahoney & Reimer
	*Chaetoceros wighamii*	Brightwell
	*Cocconeis pediculus*	Ehrenberg
	*Cocconeis placentula*	Ehrenberg
	*Cocconeis* sp.	Ehrenberg
	*Cyclotella* sp.	(Kützing) Brėbisson
	*Cylindrotheca closterium*	(Ehrenberg) Reimann & J.C.Lewin
	*Diatoma moniliformis*	Kützing
	*Diatoma tenuis*	C.Agardh
	*Diatoma vulgaris*	Bory
	*Diploneis didyma*	(Ehrenberg) Ehrenberg
	*Diploneis interrupta*	(Kützing) Cleve
	*Encyonema prostratum*	(Berkeley) Kützing
	*Entomoneis paludosa*	(W.Smith) Reimer
	*Epithemia gibba*	(Ehrenberg) Kützing 1844
	*Epithemia* sp.	Kützing
	*Fallacia* sp.	Kütz
	*Gomphonella olivacea*	(Hornemann) Rabenhorst
	*Grammatophora marina*	(Lyngbye) Kützing
	*Gyrosigma* sp.	Kützing
	*Halamphora cofeiformis*	(C.Agardh) Mereschkowsky
	*Licmophora* sp.	C.Agardh
	*Melosira moniliformis*	C.Agardh
	*Melosira nummuloides*	C.Agardh
	*Navicula gregaria*	Donkin
	*Navicula palpebralis*	Brébisson ex W.Smith
	*Navicula perminuta*	Grunow
	*Navicula ramosissima*	(C.Agardh) Cleve
	*Navicula* sp.	Bory de Saint-Vincent
	*Nitzschia sigma*	(Kützing) W.Smith
	*Opephora* sp.	Petit
	*Planothidium delicatulum*	(Kützing) Round & Bukhtiyarova
	*Pleurosigma aestuarii*	(Brébisson ex Kützing) W.Smith
	*Pleurosigma* sp.	W. Smith
	*Proschkinia poretzkajae*	(Koretkevich) D.G.Mann
	*Rhoicosphenia abbreviata*	(C.Agardh) Lange-Bertalot
	*Tabularia fasciculata*	(C.Agardh) D.M.Williams & Round
	*Tryblionella* sp.	(Grunow)
*Cyanobacteria*	*Anabaena* sp.	Bory ex Bornet & Flahault
	*Merismopedia* sp.	Meyen
	*Microcystis* sp.	Lemmermann
	*Nodularia* sp.	Mertens ex Bornet & Flahault
	*Oscillatoria* sp.	Vaucher ex Gomont
	*Spirulina major*	Kützing ex Gomont
	*Spirulina subsalsa*	Oersted ex Gomont
	*Woronichinia* sp.	A.A.Elenkin
*Myzozoa*	*Peridinium* sp.	Ehrenberg
*Chlorophyta*	*Pseudopediastrum boryanum*	(Turpin) E.Hegewald
	*Scenedesmus* sp.	Meyen
*Haptophyta*	*Prymnesium* sp.	N.Carter

**Table A2:** Average abundance and standard deviations of selected taxa.

Average Abundance	C_0	C_3	C_7	0.042_3	0.042_7	0.85_3	0.85_7	8.5_3	8.5_7
*Bacillaria paxillifera*	6550	11,033	6333	3867	2100	2900	1700	3800	1900
*Diatoma tenuis*	4050	4800	3500	1733	1750	1800	1967	1800	1250
*Melosira nummuloides*	3080	2567	5067	1950	1233	900	767	450	900
*Navicula perminuta*	3367	633	300	250	2367	700	2600	450	767
*Tabularia fasciculata*	8033	3600	2733	2533	3567	3633	2633	5033	3533
*Cylindrotheca closterium*	5233	200	100	100	400	150	100	667	700
*Entomoneis paludosa*	300	267	100	150	100	133	0	300	0
*Merismopedia* sp.	3060	1200	4200	4300	4000	5000	0	900	12,800
*Halamphora cofeiformis*	733	200	150	200	133	200	233	167	67
*Scenedesmus* sp.	1100	450	400	0	600	0	600	600	600
*Spirulina major*	83	100	100	100	100	100	100	100	0
*Spirulina subsalsa*	483	100	100	100	100	0	3400	100	0
**Standard Deviations**	**C_0**	**C_3**	**C_7**	**0.042_3**	**0.042_7**	**0.85_3**	**0.85_7**	**8.5_3**	**8.5_7**
*Bacillaria paxillifera*	476	311	200	178	66	132	70	332	98
*Diatoma tenuis*	208	147	156	146	49	72	116	170	64
*Melosira nummuloides*	201	80	297	21	110	70	64	64	121
*Navicula perminuta*	126	49	14	7	57	20	14	21	21
*Tabularia fasciculata*	93	111	85	76	133	140	85	87	188
*Cylindrotheca closterium*	283	0	0	0	14	21	0	46	0
*Entomoneis paludosa*	10	12	0	7	0	6	0	20	0
*Merismopedia* sp.	111	57	0	0	26	0	0	14	0
*Halamphora cofeiformis*	59	17	7	0	15	0	25	15	12
*Scenedesmus* sp.	99	7	0	0	28	28	0	0	0
*Spirulina major*	4	0	0	0	0	0	0	0	0
*Spirulina subsalsa*	99	0	0	0	0	0	0	0	0
